# Case of Abortion with Retained Placenta

**Published:** 1882-04-20

**Authors:** Wilmer Brinton

**Affiliations:** Independent Practitioner


### CASE OF ABORTION WITH RETAINED PLACENTA.

CASE 1st. June, 1877, Mrs A., aged 28, mother of two children, supposed herself to be near three months pregnant in June, 1877; one evening while visiting a store in vicinity of her residence, in some manner fell into an opening in the street which had been carelessly left open by some employees of the city, who were repairing and repaving the streets; her cries brought some assistance, and she was helped home; during the night she had some discharge of blood and mucus with severe pains at times which grew worse as the morning came. Dr. F. O. Donnally, now deceased, then living opposite my residence, was summoned to see her. By the time Dr. Donnally reached his patient she had aborted. The fœtus was awaiting his arrival, also the larger portion of the placenta, all of it, the doctor thought at the time, and so informed his patient that she would soon be all right; but from this time until August, a period of almost two months, his patient kept losing large amounts of blood; one day she was about, the next in bed, the doctor still being busy engaged in giving her drugs, until, as he informed me afterwards, he had given ergot and every astringent in the materia medica a fair trial.

My acquaintance with the case commenced on an excessively hot night in August, 1877, when I received a hasty summons from the doctor asking my assistance in the case. Upon my arrival there, a little after midnight, I found the woman had been having a severe hemorrhage during the afternoon and evening, which was continuing to some extent in spite of all the doctor's efforts; her pulse was 150; temp. 103½; cold sweats over her body, breathing rapid, pain over the uterus and vicinity; in fact, her general condition was alarming. Upon consultation we determined to explore the cavity of the uterus; chloroform was administered, and she was kept under the influence for over forty minutes. Upon making a digital examination I found the os dilated and dilatable, and after some slight efforts I succeeded in introducing two fingers into the cavity of the uterus, where I detected a foreign body attached to the anterior wall of the uterus, midway between the fundus and internal os, and as my fingers were considerably cramped for the want of room, it was with exertion upon my part that I was enabled to detach the mass in two pieces, one the size of a walnut, the other about the size of a peanut. After the removal of the mass I used my finger-nails as a curette, and scraped the uterine walls at the place of adhesion. The vagina and uterus were then syringed out with a weak solution of carbolic acid; at once the hemorrhage ceased; the patient rallied from the effects of chloroform nicely. I only saw this patient for two or three days following the removal of the retained and adherent placental mass, but her physician, Dr. Donnally, informed me that she had considerable metritis for ten days, but under the continued use of carbolic acid, injections of opium and quinia internally, she made a good recovery without any more hemorrhages.

CASE 2d. April, 1881, was called to see Mrs. B., a woman of the age of 40 years, of rather a fine physique, although somewhat anæmic in appearance, had several children, the youngest living being a young woman aged 17 years. My patient informed me that she had been regular until about fourteen months previous, when "she lost her period" for a month, and when they came on her again at the end of the second month she had considerable hemorrage, and passed some clots of blood. From that time until I was called to see her, a period of nearly twelve months, she had been losing a great amount of blood, not only during her regular menstrual period, but often in the interval. All this time she was still going around and attending to her household duties. Her family physician was sent for several times, during the twelve months, but, as my patient said, always made light of it, saying it was the "change of life working on her," prescribed for her, and would not return again unless sent for. To me, while she presented as specified before a somewhat anæmic appearance, I could hardly credit her account of the immense loss of blood which she had at times lost. She also complained of headache and intense pain in the back. Upon a digital examination I found the os dilated and dilatable, but not enough to introduce my finger any distance without causing some pain, so for the time desisted. Prescribed ergot gtts. xxx. and kali bromidi grs. xv. four times a day, and absolute rest in bed until I shou'd see her again within the next t.vo or three days. Just at this time the loss of blood was slight, but she informed me that just a few days previous she had an "immense" hemorrhage, that being the immediate cause of my being sent for. Two days after my seeing her I was sent for in a great hurry to see Mrs. B. again, but not being at my office at the time, it was over an hour after the message was received before I arrived at my patient's house, and found her almost in a collapsed condition, pulse hardly perceptible at the wrist, lips blue, extremities cold, and a cold sweat in large drops all over the body; in fact I found my patient as I thought in an alarming condition, the cause of all this being a hemorrhage of the most serious character, the evidence of the same being on the floor, having been in such quantities as to pass through the bddding. I found the bleeding going on to some extent, which I soon controlled by ice in the vagina, and ergot and brandy by the mouth and hypodermically, after which I tamponed the vagina with Monsel's sol. of iron diluted with water, on cotton. Two days afterwards, there being no hemorrhage in the interval, I removed this tampon and made another digital examination, and found the os much more dilated than on a previous occasion. With this examination I detected a foreign body in the cavity of the uterus, but could not get a hold of it witb my fingers. I introduced Howard's bivalve speculum, and with a long pair of placental forceps I removed a putrid and offensive mass about the size of a small lemon in the aggregate. Again introducing my finger into the uterus and satisfying myself all had been removed, syringed out the uterine cavity and vagina with a weak solution of carbolic acid, tamponed the vagina again, gave my patient grs. x. of quinia and i. gr. of opium, and ordered absolute rest for a few days. In a little over a week she was attending to her usual duties, minus some of the heavier work; has been regular ever since, and with the exception of a slight malarial attack in August, has had no need of medical attention since. No hemorrhage took place after the removal of the foreign body, and from that time the intense pain complained of in the back also disappeared to a great extent. My diagnosis in the case was a retained placenta from an abortion which took place over a year before.

CASE 3d. Was called to see Mrs. C., September, 1881, her history being as follows: Age 18, married four months. Two weeks previous to my being sent for, while washing, was taken unwell (she considering herself at this time to be about three months pregnant) large clots passed, and she went to her bed, when she had quite an extensive hemorrhage. Some hours after this a medical man of questionable repute was called in to see her, examined her, called again the next day, told her she was all right and could get up and go to work. She felt comparatively well for two or three days subsequent to this, and did resume, to some extent, her domestic duties, then had a considerable loss of blood, accompanied with some pain, which continuing, I was sent for. Upon making a digital examination found the os dilated, some tenderness in the left iliac region, pulse 100, temp. 101½, with a discharge of an offensive nature from the vagina. I prescribed for her opium, grs. ½; quinia, grs. iij., every three hours, and absolute rest in bed. The next day examined with bivalve speculum, and with my fingers and long forceps removed from the uterus without much trouble several pieces of retained placenta, amounting in the agregate to a large walnut. The uterine cavity and vagina were syringed out with a weak solution of carbolic acid for the next three or four days, and the opium and quinia continued in small doses. Within a week I discharged my patient. enjoining upon her to take all the rest possible until after her next menstrual period, and left her a tonic consisting of iron and quinia. Was again called to see her about one month afterwards, found her menses upon her, accompanied with pain, especially in the left iliac region; opium and rest were again ordered, and within a day or two all these unpleasant symptoms passed away; from then until the present time she has had no further trouble.

CASE 4th. Was called out of bed one morning in June, 1881, to see Mrs. D.; found my patient to be a large and fine-looking Irish lady, with an alarming hemorrhage from the uterus, which I soon controlled with ergot and ice in the vagina; she informed me that she was about 30 years of age, the mother of several children, all dead but one; had a miscarriage about fourteen days previous, being then nearly four months advanced in pregnancy; the abortion took place before her family physician arrived, although he had been sent for early, but was delayed in his arrival. She was kept in bed for a week, then her physician who, by the wa , is a well-known one, assured her that everything was all right, advised her to resume her usual duties, although Mrs. McN. assured me that she kept insisting to the doctor that the after-birth had never came away, but she said he laughed at her, and wanted to know "who knew best, him or her." So in this manner she was silenced, and, as mentioned before, resumed her usual duties, feeling no bad effects whatever until the morning when she had the hemorrhage, which came on her without any exertion on her part, or without any premonition of its appearance. After the hemorrhage was under control, upon an examination, I could feel the uterus through the abdominal walls, being very much larger than it ought to be in the non-pregnant state, feeling very much like a womb does after delivery at full term. Absolute rest and ergot were ordered for my patient; two days afterwards was again called out of bed to see her, and found another extensive hemorrhage going on, which I soon controlled as before, with ergot hypodermically and by the mouth, and ice in the vagina with cotton. On the following day removed the same, and found there had been considerable bleeding; introduced a bivalve speculum, and with a short pair of forceps and fingers I made my diagnosis of retained placenta, and removed several small pieces, but she complaining of pain and exhaustion, I desisted from my attempt for the day. Recognizing that I would need some assistance in the case, I called on my friend, Dr. J. H. Scarff, who responded early next morning, and on the fourth day after the hemorrhage she was placed in Sims' position, chloroformed, and Dr. Scarff and myself alternated in removing the placenta, it being done by introducing the hand into the vagina and finger or fingers into the cavity of the uterus, and removing the placenta (which we found firmly attached to the walls of the uterus) in pieces. Judging from the amount of placenta removed, I would suppose the woman as right in believing herself four months pregnant when the abortion took place, and why this large placenta escaped the notice of her medical attendant is beyond my comprehension. After the removal of the placenta the parts were syringed with a weak solution of carbolic acid and the vagina tamponed, but from this time there was no more hemorrhage, but within thirty-six hours a severe chill occurred, followed with high fever, the temperature being 105½, pulse 150, and my patient had a severe attack of metritis and cellulitis, and she was alarmingly ill for two weeks, but thanks to about 800 grs. of quinia and nearly a drachm of opium, taken during the time of her illness, she made a beautiful recovery, and has recently expressed to me that she was free from pains and aches, and had better health than she had had for years.

From the report of the cases under consideration (my line of treatment having been often mentioned) I make it an axiom: in retained placenta, with hemorrhage or not, remove the cause and treat the symptoms as they arise.

From my own experience with these and other cases coming under my notice, I have been impressed with the following facts:

1st. Their frequency.

2d. That a hemorrhage with a dilated and dilatable os, as a rule, means a foreign body in the cavity of the uterus.

3d. That in opium and quinia we have almost a specific (if given in proper doses) in controlling inflammation of the uterus and its appendages, setting up after operative interference with the same.

4th. That in the fingers we have a valuable instrument in removing the foreign bodies from the cavity of the uterus.

5th. The wonderful recuperative powers of woman after the loss of an immense amount of blood.

6th. As long as we have a dilated os, medicated injections into the cavity of the uterus are harmless, and in them we have a valuable aid in the treatment of the class of cases under consideration, especially subsequent to the removal of the retained placenta.

In conclusion, I would say that if I have succeeded in impressing on the minds of the members of this society the necessity of watching their patients after abortion, especially if there is a suspicion of retained placenta, this suspicion being verified or not before discharging them, either by digital or other forms of examination; I say sir, if I have succeeded in doing this, one of the main objects of my paper has been accomplished.—*Dr. Wilmer Brinton*, *Independent Practitioner*.

